# Molecular glue degraders: Rational design, specificity engineering, and advanced delivery

**DOI:** 10.1016/j.apsb.2026.02.020

**Published:** 2026-03-02

**Authors:** Lieen Ma, Ning Wang, Jingjing Zhu, Lingjie Wu, Shan He, Bin Zhang

**Affiliations:** a School of Pharmacy, Health Science Center, Ningbo University, Ningbo 315211, China; b Institute of Drug Discovery Technology, Ningbo University, Ningbo 315211, China; c Research & Development Center, HEALTH BioMed Co., Ltd., Ningbo 315040, China; d Ningbo Institute of Marine Medicine, Peking University, Ningbo 315832, China

**Keywords:** Molecular glue degraders, Targeted protein degradation, Rational design, E3 ligases, Specificity engineering, Drug delivering systems

## Abstract

Molecular glue degraders (MGDs) have emerged as a transformative modality in the field of targeted protein degradation (TPD), enabling the selective elimination of disease-relevant proteins, including those traditionally considered undruggable. Unlike bifunctional proteolysis-targeting chimeras (PROTACs), MGDs operate through monovalent architectures that induce protein–protein interactions (PPIs) between E3 ligases and neosubstrates, offering advantages in chemical simplicity, cell permeability, and target scope. However, MGD discovery remains serendipitously, and a translational framework that links rational design to predictable selectivity and tissue exposure is still lacking. In this review, we present an integrated framework for advancing next-generation MGDs through three critical dimensions: rational design, specificity optimization, and delivery systems. First, we examined cutting-edge strategies in MGD design, including covalent handle-based reprogramming, PPI-driven stabilization, and multi-site, multi-functional constructs. Second, we explored structure-guided engineering and chemoinformatic models, such as cereblon degron motifs, zone-based design and multiparameter optimization, to improve neosubstrate selectivity while minimizing off-target liabilities. Third, we summarized delivery platforms, including antibody‒drug conjugates, nanoparticle-enabled systems, and folate-mediated targeting, which are primarily intended to improve tissue selectivity and targeted distribution, thereby promoting local tissue accumulation. Finally, we discussed emerging opportunities at the intersection of artificial intelligence, structural biology, and systems pharmacology for accelerating MGD discovery and clinical translation. Collectively, these interdisciplinary insights underscore the therapeutic promise of MGDs and lay the groundwork for their next-generation evolution in precision medicine.

## Graphical abstract

This review summarizes emerging strategies for molecular glue degraders, highlighting rational design principles, specificity engineering, and advanced delivery platforms that collectively accelerate selective degradation of undruggable proteins for precision medicine.

**Figure:**
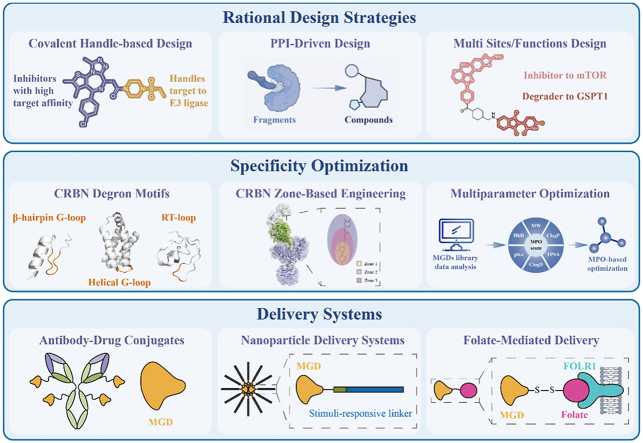

## 1 Introduction

**Figure 1:**
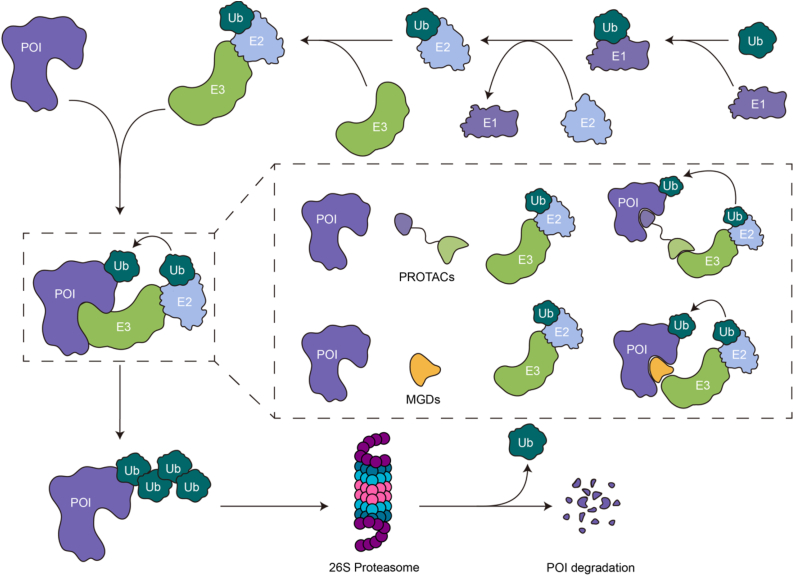
Mechanistic comparison of targeted protein degradation by heterobifunctional PROTACs *versus* monovalent molecular-glue degraders (MGDs). Both modalities recruit an E3 ligase to the protein of interest (POI) to trigger polyubiquitination and 26S-proteasomal clearance. PROTACs tether the POI and E3 with a linker, whereas MGDs stabilize a direct POI-E3 interface.

**Figure 2:**
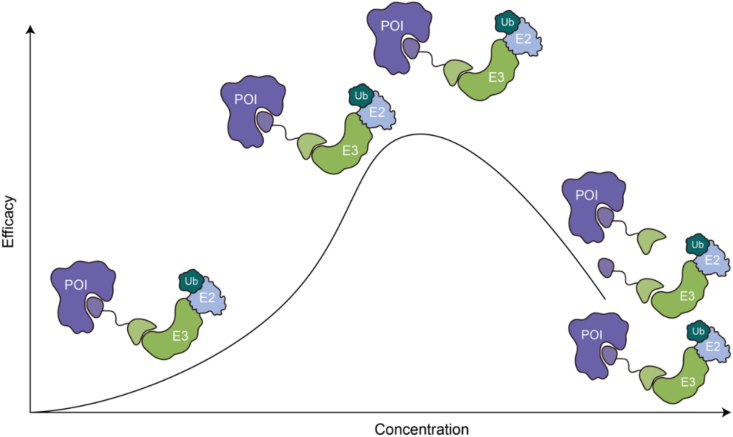
Biphasic concentration-efficacy (“hook”) profile of PROTAC-mediated degradation. Increasing doses initially favor productive ternary POI-PROTAC-E3 assemblies, boosting ubiquitination. However, at supra-stoichiometric concentrations, excess PROTAC shifts the equilibrium toward non-productive binary complexes, dampening degradation.

The ubiquitin–proteasome system (UPS) plays a central role in maintaining cellular protein homeostasis by mediating the recognition and degradation of specific proteins *via* E3 ubiquitin ligases^1^. In recent years, targeted protein degradation (TPD) has emerged as a groundbreaking strategy in drug discovery, which leverages small molecules to redirect the ubiquitin–proteasome pathway toward eliminating disease-associated proteins^2^^,^^3^. Among the various TPD strategies, molecular glue degraders (MGDs) are linker-less compounds that promote protein‒protein interactions (PPIs) between E3 ligases and proteins of interest (POIs)^4^. Mechanistically, MGDs function by binding to an E3 ligase and altering its surface properties, thereby creating a novel protein-interaction interface or stabilizing weak endogenous interactions, and consequently recruiting otherwise non-interacting substrates into close proximity with the ligase catalytic domain. This induced proximity facilitates substrate ubiquitination and subsequent proteasomal degradation. Unlike MGDs, bifunctional proteolysis-targeting chimeras (PROTACs) employ two distinct binding moieties, one targeting an E3 ligase and the other engaging the POI, linked by a chemical tether to facilitate their proximity (Fig. 1)^4^. Molecular glues generally exhibit a linear dose-response profile, whereas PROTACs exhibit a “hook effect”, a phenomenon where excessive concentration of bifunctional degraders leads to decreased efficacy due to the formation of non-productive binary complexes (Fig. 2).

Owing to their unique mechanisms of action, MGDs and PROTACs offer significant advantages over traditional small-molecule inhibitors, particularly in targeting proteins lacking well-defined binding pockets, such as transcription factors, which have long been deemed “undruggable”. For instance, lenalidomide, a molecular glue that enhances cereblon-mediated degradation of specific transcription factors, has demonstrated remarkable efficacy in treating multiple myeloma^5^, ^6^, ^7^. Historically, MGDs, such as immunomodulatory imide drugs (IMiDs) (*e.g.*, thalidomide and lenalidomide), were identified through phenotypic screening, with their function as inducers of E3 ligase–substrate interactions clarified subsequently^8^. Although recent advances in chemo-proteomics and high-throughput screening have facilitated a more rational drug discovery process, designing MGDs with expanded therapeutic potential and improved clinical safety remains a major challenge^9^.

Nevertheless, given their potential to modulate previously intractable targets, MGDs represent a promising avenue for precision medicine. While IMiDs have significantly improved outcomes in multiple myeloma, recent studies have explored alternative E3 ligases, such as KEAP1, which broaden the therapeutic applications of MGDs, including the treatment of oxidative stress-related neurodegenerative disorders^10^. Additionally, emerging MGDs are under investigation for hematological disorders, such as sickle cell disease^11^^,^^12^, and immunological conditions. Consequently, the ongoing discovery of novel MGDs targeting diverse E3 ligases, such as DCAF15 and KEAP1, along with an expanding repertoire of substrates (Table 1), underscores the need for a comprehensive review to synthesize current advancements and guide future developments in this dynamic field.

**Table 1:** Catalog of molecular glue degraders: E3 ligases, neo-substrates, and representative compounds.

E3 ligase	Target	MGD examples	Ref.
CRBN	ARID2	Pomalidomide	13
CRBN	CDK2	MRT-51443, PLX-513	14,15
CRBN	CK1ɑ	Lenalidomide, TMX-4116	16, 17, 18
CRBN	Cyclin E1 (CCNE1)	MRT-50969	19
CRBN	ENL	rac-dHTC1	20
CRBN	GSPT1	CC-885, CC-90009	21
CRBN	IKZF1, IKZF3	Pomalidomide, Lenalidomide	7,22,23
CRBN	IKZF2	NVP-DYK709	24
CRBN	NEK7	MRT-3486	25
CRBN	PDE6D	TMX-4100	18
CRBN	SALL4	Thalidomide	26,27
CRBN	VAV1	MRT-23227	25
CRBN	WIZ	dWIZ-1/2	12
CRBN	ZMYM2	CC-122	28
CRBN	ZFP91	CC-92480	29
DCAF11	BRD4	IBG4	30
DCAF15	RBM23, RBM39	Indisulam, E7820, CQS	31, 32, 33
DCAF16	BRD4	IBG1	30
DCAF16	BRD4	JP-2-197	34
DDB1	CyclinK	CR8, dCEMM2/3/4, HQ461	35, 36, 37
KEAP1	NRF2	VVD-065	38
KEAP1	EGFR	CDDO-Me	10
RNF4	BRD4	CCW 28-3	39
RNF114	BRD4	XH2 (nimbolide-JQ1)	40
RNF114	BCR-ABL	XH2 (nimbolide-dasatinib)	41
RNF126	CDK4	EST1027	42
RNF126	BTK	JP-2-196+BTK ligand	42
RNF126	BCR-ABL	JP-2-196+dasatinib ligand	42
SIAH1	BCL6	BI-3802	43,44
VHL	CDO1	Cpd4, Cpd8	45
*β*-TrCP	*β*-Catenin	NRX-252114	46

Therefore, this review provides a comprehensive framework for the rational design of MGDs, beginning with mechanistic strategies that include covalent handle-based modification, PPI-driven design, and multi-site, multi-functional approaches. Subsequently, we examined structure-guided and chemoinformatic methods to enhance selectivity and safety, with a focus on cereblon-based degraders. In parallel, we explored advanced delivery modalities, including antibody‒drug conjugates (ADCs), nanoparticle platforms, and folate-mediated systems, which are shaping the clinical translation of MGDs. Finally, we outlined emerging opportunities at the intersection of artificial intelligence, structural biology, and degradation prediction, highlighting how next-generation computational platforms may redefine the discovery and optimization of MGDs.

## 2 Rational design strategies in MGDs

**Figure 3:**
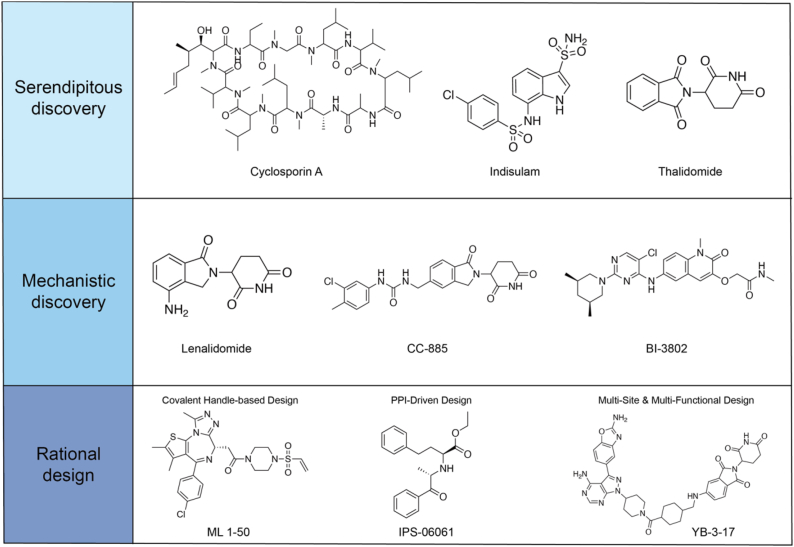
Conceptual evolution of molecular-glue degrader (MGD) discovery. Serendipitous findings (cyclosporin A, indisulam, thalidomide) were followed by mechanistic screens that revealed ligase-recruiting chemotypes (lenalidomide, CC-885, BI-3802). In contrast, recent structure-guided efforts achieved rationally designed MGDs (ML 1-50, IPS-06061, YB-3-17). Chemical structures of representative compounds are depicted in the figure.

The concept of molecular glues originated in the early 1990s with the serendipitous discovery of immunosuppressants such as cyclosporin A (CsA), FK506, and rapamycin, which exert their effects by stabilizing protein‒protein interactions (PPIs)^47^^,^^48^. CsA and FK506, for instance, act by forming ternary complexes with calcineurin only when bound to their respective chaperones, cyclophilin and FKBP. Similarly, rapamycin facilitates FKBP-mediated targeting of mTOR through induced complex formation. Notably, these classical immunophilin-dependent compounds are now widely viewed as non-degrading, stabilizing molecular glues that modulate protein function by reinforcing specific PPIs rather than inducing proteasomal clearance. This stabilizing-glue direction has rapidly expanded through foundational contributions from the Schreiber, Arkin, and Waldmann groups and recent perspectives in the field^48^, ^49^, ^50^. In parallel, serendipitously discovered degradative glues, such as indisulam and thalidomide, exemplify early empirical entry points into molecular glue degraders. These initial findings, along with the phenotypic identification of immunomodulatory drugs (IMiDs) like lenalidomide, laid the foundation for molecular glue degraders (MGDs) as a drug discovery paradigm. However, such discoveries were largely empirical and offered limited control over specificity, efficiency, or substrate scope (Fig. 3).

Subsequent mechanistic discoveries involving lenalidomide and other compounds, such as CC-885 and BI-3802, advanced the understanding of target‒ligase recognition and ternary complex formation, greatly contributing to the mechanistic understanding of MGDs. These discoveries marked a significant transition from purely empirical findings to a deeper mechanistic comprehension.

Recent advances in structural biology, chemoproteomics, and high-throughput screening have catalyzed a shift from empirical screening to rational MGD design^9^^,^^37^^,^^51^^,^^52^. In this section, we systematically examine three emerging design strategies: (1) covalent handle-based chemical modification, (2) PPI-driven interface stabilization, and (3) multi-site and multi-functional constructs. These approaches collectively represent a modular design toolkit for expanding the functional landscape and therapeutic potential of MGDs.

### 2.1 Covalent handle-based design

**Figure 4:**
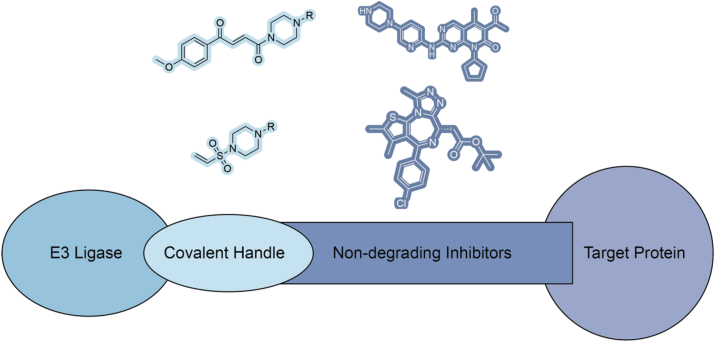
Covalent-handle strategy for turning non-degrading inhibitors into molecular-glue degraders (MGDs). Representative electrophilic handles (top) are appended to inhibitor scaffolds, yielding bifunctional molecules that bridge an E3 ubiquitin ligase (left) and the target protein (right) to trigger proximity-induced ubiquitination and degradation.

To move beyond serendipitous discovery, rational design strategies focus on structural optimization to transform non-degrading inhibitors into effective MGDs. This approach utilizes covalent modifications, such as electrophilic warheads, to induce ubiquitination and proteasome-mediated degradation, thereby expanding the therapeutic potential of TPD. Recent studies by the Nomura group^34^^,^^42^ have demonstrated the utility of covalent handles as versatile tools in MGD design. These handles, which incorporate electrophilic groups such as fumarate derivatives and vinylsulfonyl piperazine, enable stable interactions with E3 ligases, thereby facilitating the conversion of inhibitors into MGDs. Their flexibility allows them to engage a diverse range of neo-substrates and E3 ligases, such as DCAF16 and RNF126, thus broadening the scope of applicable MGDs and reducing the reliance on serendipitous discoveries (Fig. 4).

Fumarate derivatives, such as but-2-ene-1,4-dione, exemplify the versatility of covalent handles in transforming the CDK4/6 inhibitor ribociclib into molecular glue degraders. EST1027, which incorporates a trifluoromethylphenyl cinnamamide handle, achieved >50% CDK4 reduction at 3 μmol/L but lacked generalizability across similar CDK4/6 inhibitors such as palbociclib. In contrast, EST1060, featuring a fumarate handle, demonstrated enhanced degradation efficiency through stronger engagement with RNF126. Beyond CDK4, this handle enabled the degradation of diverse neo-substrates, including BRD4, BCR-ABL, and PDE5, primarily *via* RNF126, with potential contributions from additional RING-family E3 ligases, such as RNF40, MID2, RNF219, RNF14, and LRSAM1, as suggested by chemoproteomics profiling^42^. Fumarate-based covalent degraders not only induce BCR-ABL degradation^42^^,^^53^ but also facilitate RNF126-mediated Lin28 degradation^54^, demonstrating their broad applicability in expanding the scope of MGDs *via* multiple E3 ligase pathways.

Vinylsulfonyl piperazine, another key covalent handle, has been incorporated into BET family inhibitors such as JQ1, enabling BRD4 degradation through covalent bonding with DCAF16's Cys119, which facilitates substrate recruitment for proteasomal degradation. This handle also enabled the DCAF16-dependent degradation of CDK4, SMARCA2/4, and BCR-ABL/c-ABL. Although AR degradation was observed, its dependence on DCAF16 remains uncertain, as viability issues in LNCaP cells precluded definitive assessment. These findings underscore the versatility of this covalent handle in converting various non-degrading inhibitors into effective monovalent degraders. While this expands the potential applications of DCAF16-based molecular glue strategies, the selectivity of degradation varies among targets, necessitating further refinement^34^.

**Figure 5:**
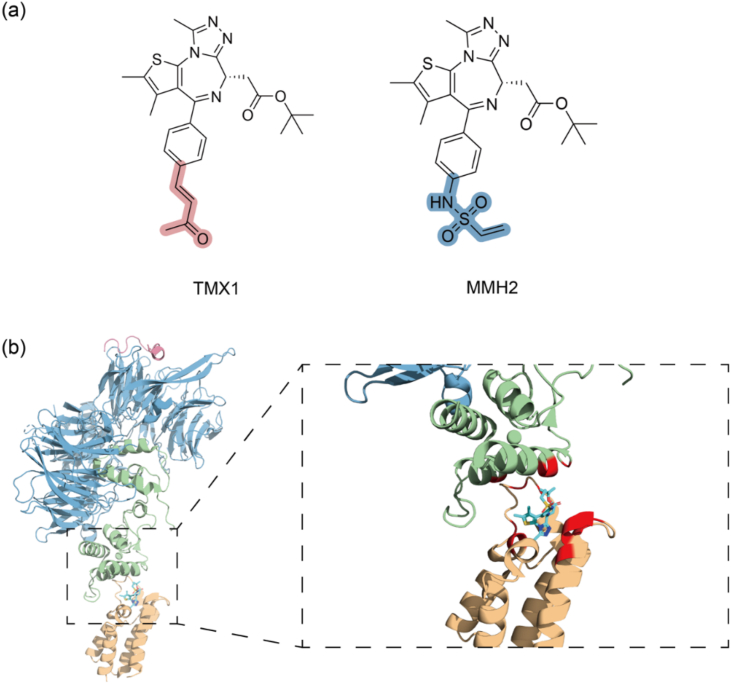
Template-assisted covalent modification and warhead optimization. (a) Chemical structures of covalent molecular glue degraders (MGDs) TMX1 (pink, *α*,*β*-unsaturated aldehyde warhead) and MMH2 (blue, sulfonyl fluoride warhead). The electrophilic warheads, highlighted in color, were optimized to enhance covalent binding specificity and potency. (b) Crystal structure illustrating the ternary complex: DCAF16 (green), BRD4 (tan), and the CRL4 E3 ligase complex (blue) are bridged by MMH2 (cyan). The inset highlights covalent bond formation between the electrophilic warhead of MMH2 and a cysteine residue (red) in DCAF16, which stabilizes the ternary complex and promotes efficient ubiquitination and degradation of BRD4 (PDB: 8G46).

Template-assisted covalent modification and warhead optimization refine covalent handles by enhancing target specificity and degradation efficiency. By fine-tuning the electrophilic reactivity and chemical properties of warheads, these approaches precisely control covalent interactions, thus ensuring selective and robust degradation (Fig. 5). For example, structural complementarity between BRD4 and DCAF16, promoted by strategically positioned covalent warheads, such as TMX1 and MMH2, stabilizes ternary complexes and significantly enhances BRD4 degradation potency and selectivity^55^.

This strategy also shows potential applicability toward other neo-substrates, such as GAK, although its effectiveness might be influenced by factors including cellular context, substrate abundance, and specific E3 ligase availability. Optimization of warhead reactivity and structural complementarity reduces off-target interactions and enhances degradation specificity. Continued structure-guided refinement of covalent handles may further extend their versatility, ultimately advancing the broader development of molecular glue degraders with improved efficacy and safety profiles^55^.

Despite these advancements, covalent handle-based MGDs still face intrinsic challenges, with cytotoxicity being a major concern^53^. Cytotoxicity arises from the electrophilic nature of covalent handles, which can lead to off-target reactivity and unintended protein modifications. For example, *α*,*β*-unsaturated carbonyls and sulfonyl fluoride warheads may react indiscriminately with nucleophilic residues in non-target proteins, resulting in cellular stress and toxicity^56^. To mitigate this issue, warhead optimization strategies such as biorthogonal chemistry, steric shielding, and reactivity modulation have been proposed to improve selectivity while maintaining degradation efficiency^55^^,^^56^.

The chemical modification strategy provides a versatile approach for transforming non-degrading inhibitors into potent MGDs^36^. Covalent handles, such as fumarate derivatives and vinylsulfonyl piperazine, have broadened the applicability of TPD^30^^,^^34^^,^^37^, serving as alternatives to PROTACs by enabling interactions with diverse E3 ligases and neo-substrates^9^^,^^39^. However, discovering novel covalent handles remains challenging, as their rational design requires an in-depth understanding of protein‒ligand interactions and E3 ligase selectivity^39^. Future research should focus on systematically identifying new chemical modification modules and elucidating their degradation mechanisms to refine MGD design^57^. Given these challenges, a systematic strategy to discover novel covalent handles with enhanced specificity and efficiency is essential^58^. By integrating chemical screening, quantitative proteomic analyses, and mechanistic validations, researchers can address these limitations and generate more predictable and robust MGD scaffolds^9^^,^^39^^,^^40^. Such a systematic discovery framework will further refine covalent handles, enabling more predictable and highly selective protein degradation outcomes.

**Figure 6:**
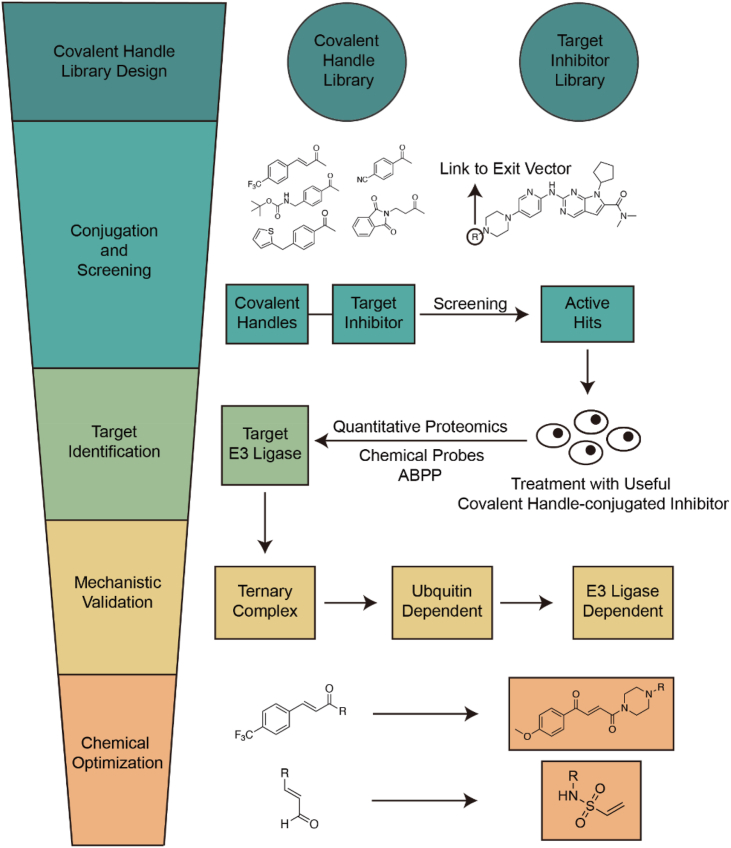
Workflow for discovering covalent-handle molecular-glue degraders (MGDs). Electrophilic handles are appended *via* a modular exit vector to a target–inhibitor library and screened in cells. Activity-based protein profiling (ABPP)-guided quantitative proteomics reveals the recruited E3 ligase, and ligase-knockout validation confirms dependency, guiding medicinal-chemistry optimization of handle and linker.

To systematically develop universal covalent handles for TPD, an integrated discovery framework is essential. This approach involves a series of well-defined steps, from initial chemical library design to functional validation and optimization (Fig. 6).

The initial step involves the construction of a comprehensive covalent handle library comprising diverse electrophilic warheads, designed to selectively react with modifiable amino acid residues^37^^,^^39^. These handles are conjugated with inhibitors that target specific POIs, thereby facilitating the systematic evaluation of their degradation potential. Subsequently, quantitative proteomics is employed to map the protein targets and elucidate the specificity and underlying degradation mechanisms associated with each handle^9^. This is followed by probe-based chemoproteomics is then applied to capture proteins interacting with the covalent handle, facilitating the identification of candidate E3 ligases^39^^,^^40^. Activity-based protein profiling (ABPP) further pinpoints specific amino acid residues within E3 ligases that serve as covalent binding sites^39^.

Thereafter, validation of candidate E3 ligases is conducted *via* CRISPR-mediated knockout or RNAi knockdown experiments^9^. In parallel, proteasome inhibitors, such as MG132, are used to determine whether degradation is dependent on the UPS. Loss of degradation in E3 ligase-deficient cells or in the presence of proteasome inhibitors confirms pathway dependency.

Subsequent optimization refines the chemical properties of covalent handles, further enhancing their degradation efficiency and selectivity, while simultaneously minimizing off-target effects^34^. Finally, the versatility of the optimized handle is assessed by conjugating it to multiple inhibitors to evaluate its generalizability across different protein targets^30^^,^^37^. This systematic workflow provides a rational approach to developing universal covalent handles, thereby broadening the scope of MGDs.

### 2.2 PPI-driven design

Unlike covalent handle-based strategies, the PPI-driven design approach leverages intrinsic binding interfaces between E3 ubiquitin ligases and neo-substrates to construct complementarity between molecular surfaces. This strategy is particularly powerful in targeting conventionally “undruggable” proteins, such as KRAS G12D and VAV1, which lack accessible small-molecule binding pockets^59^^,^^60^.

KRAS is one of the most frequently mutated oncogenes across human cancers, with KRAS G12D mutations being highly prevalent in pancreatic ductal adenocarcinoma (PDAC), colorectal cancer (CRC), and non-small cell lung cancer (NSCLC)^61^^,^^62^. This mutation results in constitutive activation of the RAS-MAPK signaling pathway, promoting uncontrolled cell proliferation and tumor progression^63^. KRAS G12D has historically been considered an “undruggable” target due to its high-affinity GTP/GDP-binding pocket and the lack of suitable allosteric sites, presenting significant hurdles in therapeutic development^64^.

A recent breakthrough in addressing this challenge is the development of IPS-06061, a novel MGD specifically designed to induce KRAS G12D degradation *via* CRBN-mediated ubiquitination. IPS-06061 acts by forming a stable ternary complex with cereblon (CRBN), the substrate receptor within the CRL4 E3 ubiquitin ligase complex, and KRAS G12D, leading to selective ubiquitination and subsequent proteasomal degradation of the mutant protein^59^. This achievement exemplifies how PPI-based strategies, integrating computational modeling and fragment-based drug design, can overcome the inherent limitations in targeting difficult oncogenic proteins.

The rational design of IPS-06061, disclosed in patent WO2024241248, was initiated with the deliberate selection of CRBN as the recruiting E3 ligase due to its well-defined structure, proven druggability with thalidomide analogs, and broad application in targeted protein degradation^65^. Structural modeling and interaction prediction relied on a recombinantly expressed His-tagged CUL4A-RBX1-DDB1-CRBN complex (a reconstituted CRL4 E3 ligase comprising Cullin 4A, RING-box protein 1, DDB1, and Cereblon), laying the groundwork for subsequent computational analysis. The use of computational tools, such as Maestro (Schrödinger), AutoDock Vina, and NAMD, was central to this approach to simulate and optimize ternary complex formation between CRBN, KRAS G12D, and potential molecular glues^59^.

**Figure 7:**
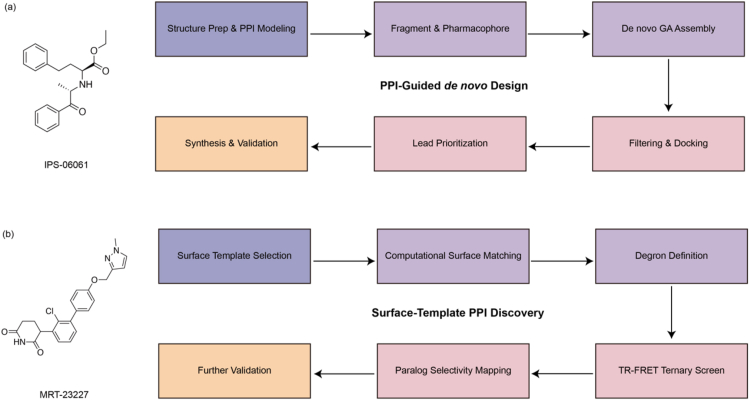
Workflows for PPI-driven design of molecular-glue degraders. (a) PPI-guided *de novo* design of a CRBN molecular glue degrader for KRAS G12D (IPS-06061). (b) Surface-template PPI discovery of a CRBN molecular glue degrader for VAV1 (MRT-23227).

Given the absence of viable allosteric binding sites on KRAS G12D, the design strategy prioritized orthosteric interactions, targeting small molecules capable of directly stabilizing the CRBN-KRAS G12D interface. Subsequently, a fragment-based drug discovery workflow was employed (Fig. 7), starting with the identification of small, drug-like fragments displaying favorable interactions with either KRAS G12D or CRBN. Pharmacophore modeling in Maestro mapped critical interaction motifs, including hydrogen bond donors and acceptors, and hydrophobic interactions, within the PPI interface, which served as initial “seeds” for *de novo* molecular generation using a genetic algorithm implemented through the LEASD server. The algorithm applied Lipinski's Rule of Five criteria to ensure candidate molecules possessed favorable drug-like properties (molecular weight <500 Da, optimal lipophilicity, and good oral bioavailability)^66^. Top-ranked candidate molecules were selected based on docking scores and predicted binding stability within a 15 Å search radius around the interface. Ultimately, this rigorous process identified IPS-06061 as the precursor compound, which was subsequently validated experimentally for its capacity to induce robust, CRBN-dependent degradation of KRAS G12D^59^.

VAV1 provides another compelling example of PPI-driven molecular glue design in cases where the target protein is undruggable, and was genetically validated and broadly implicated in autoimmune and chronic inflammatory diseases^67^^,^^68^. However, VAV1 remains a difficult drug target because it functions as a guanine-nucleotide exchange factor (GEF) for Rho-family GTPases, and none of its seven folded domains is predicted to contain G-loop motifs^69^, ^70^, ^71^. Accordingly, the PPI-driven objective for targeting VAV1 is to use the intrinsic binding interfaces to stabilize the productive CRBN-molecular glue-target ternary complex by engineering complementarity between molecular surfaces.

Recently, Monte Rosa Therapeutics implemented this concept by treating established CRBN degron/neosubstrate interfaces as structural “degron-surface templates” (*e.g.*, the GSPT1 interface in the presence of CC-90009) and applying computational surface-matching algorithms to search for analogous surface patches across VAV1 domains^25^. This workflow identified a candidate patch within the VAV1 C-terminal SH3 domain (SH3c) that recapitulates key shape/electrostatic features of the GSPT1-CRBN engagement surface despite the absence of underlying fold or motif homology. Subsequently, they used the predicted SH3c patch as a “bait” in an orthogonal ternary-complex assay (TR-FRET) to screen an internal compound library and identified MRT-23227 as a potent ternary-complex inducer.

Notably, MRT-23227 exhibited pronounced selectivity for VAV1 over its close paralogues VAV2 and VAV3, despite their high overall sequence and domain architecture similarity. This selectivity was attributed to subtle, non-conserved surface features within the RT-loop part. Recruitment of full-length VAV1 was then confirmed in cells by NanoBRET, and CRBN-dependent VAV1 degradation was demonstrated using a NanoBiT-based degradation reporter system, establishing a closed loop from *in silico* interface discovery to cellular function. Mechanistically, the identification of MRT-23227 further illustrates how PPI-driven molecular glues can achieve both target engagement and paralogue selectivity without reliance on canonical degron motifs (Fig. 7b).

Collectively, the examples of KRAS G12D and VAV1 highlight the feasibility of PPI-driven molecular glue design, and underscore its particular suitability for targets that lack well-defined small-molecule binding pockets. Complementary interaction surfaces can be engineered through pharmacophore definition, library construction, and high-throughput screening to promote the recruitment of target proteins to E3 ubiquitin ligases, enabling ubiquitination-dependent degradation. Notably, target recruitment and ternary complex formation alone do not guarantee efficient degradation; rather the iterative optimization of compound structures is often required to convert initially “non-productive” recruitment into degradable, functionally competent ternary complexes.

### 2.3 Multi-site & multi-functional design in chemical inducers of proximity (CIPs)

Single-site MGDs have significantly advanced targeted protein degradation by selectively stabilizing PPIs to induce degradation of disease-relevant targets. However, single-site designs can be constrained by limited ternary-complex stability, vulnerability to resistance mutations, and the lack of tractable pockets on certain targets^72^, ^73^, ^74^. Therefore, some recent studies are exploring multi-site and multi-functional chemical inducers of proximity (CIPs). These strategies are broadly classified into the following categories.

#### 2.3.1 Single-site/dual-function CIPs

**Figure 8:**
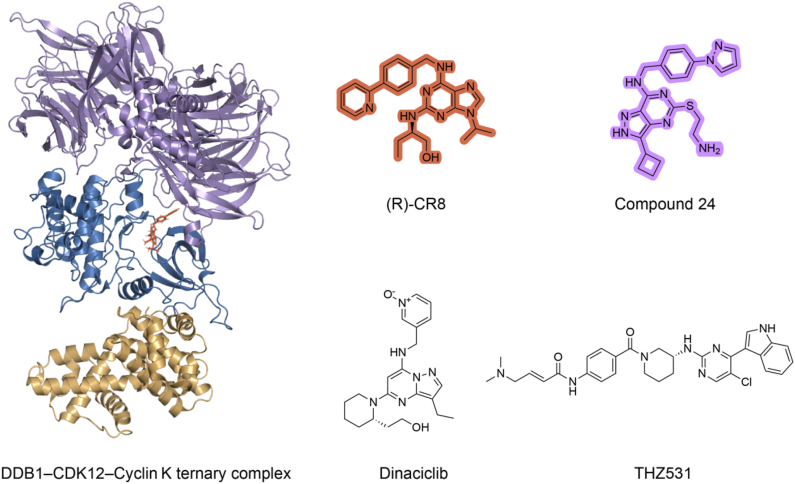
Structural and chemical comparison of CDK12-targeting inhibitors *versus* single-site dual-function molecular-glue degraders (MGDs). (Left) Crystal structure of the DDB1-CDK12-Cyclin K ternary complex stabilized by (*R*)-CR8 (orange sticks) (PDB: 6TD3). Chains are colored as follows: Cyclin K (violet), CDK12 (blue) and DDB1 (sand). (Top right) Chemical structures of the two validated single-site dual-function MGDs, (*R*)-CR8 and the higher-potency Compound **24**, both of which inhibit CDK12 and induce Cyclin K degradation by recruiting the DDB1-CUL4 E3 ligase. (Bottom right) Reference ATP-site inhibitors that lack degradation activity, Dinaciclib (pan-CDK) and THZ531 (covalent CDK12/13), are shown for contrast.

Compound **24**, a 3,5,7-trisubstituted pyrazolo[4,3-*d*]pyrimidine, represents a significant advancement in single-site dual-function MGDs, which integrates CDK12 inhibition with Cyclin K degradation^75^. Unlike conventional ATP-competitive CDK inhibitors (*e.g.*, dinaciclib^76^^,^^77^) or covalent CDK12 inhibitors such as THZ531^78^, that selectively inhibit CDK12 kinase activity but fail to induce Cyclin K degradation, Compound **24** exhibits an additional molecular glue mechanism. Compound **24** stabilizes the CDK12–DDB1 interaction, thereby facilitating recruitment of the DDB1-CUL4 E3 ligase complex, leading to Cyclin K ubiquitination and subsequent proteasomal degradation. This degradation mechanism resembles that of CR8, a previously characterized dual-function molecular glue that acts as a CDK12 inhibitor and a Cyclin K degrader^35^^,^^75^. However, compared to CR8, Compound **24** demonstrates over a 100-fold greater potency in reducing Cyclin K levels in MINO lymphoma cells, as confirmed by Western blot analysis^75^ (Fig. 8). This enhanced efficacy is attributed to stronger CDK12 binding affinity and improved DDB1 recruitment, establishing Compound **24** as a highly potent single-site dual-function degrader.

#### 2.3.2 Dual-site/dual-function CIPs

**Figure 9:**
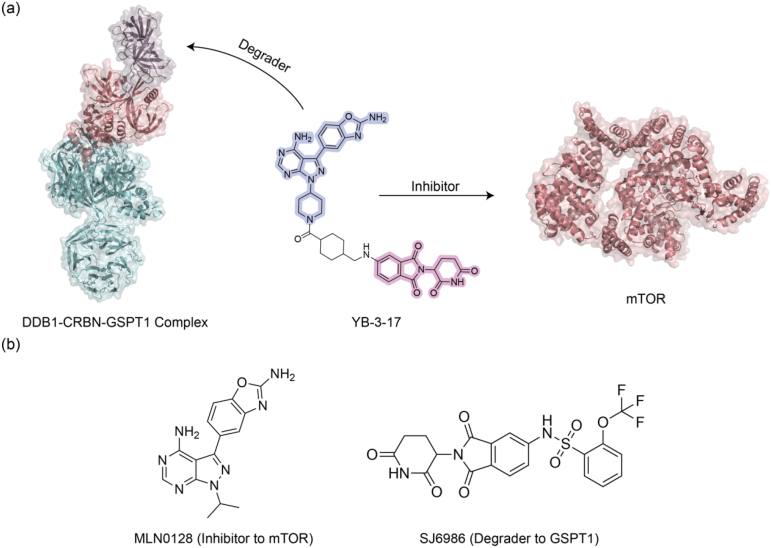
Structural and mechanistic representation of YB-3-17 as a dual-site, dual-function degrader targeting mTOR and GSPT1. (a) YB-3-17 is a rationally designed small molecule that combines two distinct pharmacophores: one moiety (purple) binds and inhibits the ATP-binding site of mTOR (right, PDB: 4JSV), while the other (blue) recruits the E3 ligase substrate receptor cereblon (CRBN) to promote the degradation of GSPT1 (left, PDB: 5HXB). YB-3-17 simultaneously inhibits mTOR kinase activity and induces CRBN-mediated GSPT1 ubiquitination, thereby achieving enhanced antitumor efficacy through dual-target disruption. (b) Chemical structures of the reference compounds MLN0128 (a selective mTOR inhibitor) and SJ6986 (a cereblon-based degrader of GSPT1), which were used for comparative analysis of the dual mechanism of action of YB-3-17.

YB-3-17 is a dual-target, dual-mechanism (DTDM) small molecule designed to simultaneously inhibit mammalian target of rapamycin (mTOR) and degrade G1 to S phase transition 1 (GSPT1), to enhance antitumor efficacy and overcome the limitations of monotherapy^79^. mTOR plays a central role in the PI3K–AKT–mTOR signaling pathway, and its aberrant activation is closely linked to glioblastoma (GBM) progression. However, conventional mTOR inhibitors have demonstrated limited clinical success due to drug resistance, suboptimal selectivity, and adverse side effects^80^^,^^81^. GSPT1 is a translation termination factor overexpressed in various cancers, and its targeted degradation has been shown to suppress tumor cell proliferation^82^. Through iterative, hypothesis-guided optimization and screening, including structural optimization, the authors revealed YB-3-17 as a molecule that simultaneously inhibits mTOR and induces GSPT1 degradation (Fig. 9). In U87 glioblastoma cells, YB-3-17 exhibited high antiproliferative activity (IC_50_ = 3.3 nmol/L), significantly outperforming the mTOR inhibitor MLN0128 and the GSPT1 degrader SJ6986^79^. Molecular dynamics simulations revealed that YB-3-17 stabilizes its interaction with mTOR *via* a hydrogen bond with Lys2306, while promoting CRBN-mediated GSPT1 degradation (DC_50_ = 5 nmol/L)^79^. Further *in vivo* studies confirmed that YB-3-17, at doses of 10‒20 mg/kg, almost completely suppressed tumor growth, exhibiting lower toxicity compared to MLN0128 alone^79^. Additionally, RNA-sequencing analysis showed that YB-3-17 effectively downregulates the mTORC1/mTORC2 signaling and modulates p53 and apoptosis-related gene expression, highlighting its potential to overcome drug resistance^83^. Additionally, the successful development of YB-3-17 validates the feasibility of the DTDM strategy, offering a promising framework for next-generation anticancer drug design that integrates small-molecule inhibition and targeted protein degradation^79^.

Additionally, a promising subclass of dual-site molecular glues has emerged that adopts a dual-degradation strategy, simultaneously directing two distinct neosubstrates towards ubiquitin-proteasome-mediated elimination. This approach is particularly advantageous in complex disease contexts, such as hematologic malignancies, where concurrent disruption of parallel oncogenic pathways may be necessary to achieve durable therapeutic responses. Two representative examples of this strategy are KT-413 and GBD-9, both of which integrate PROTAC and molecular glue mechanisms within a single molecular architecture to achieve synergistic dual-target degradation.

**Figure 10:**
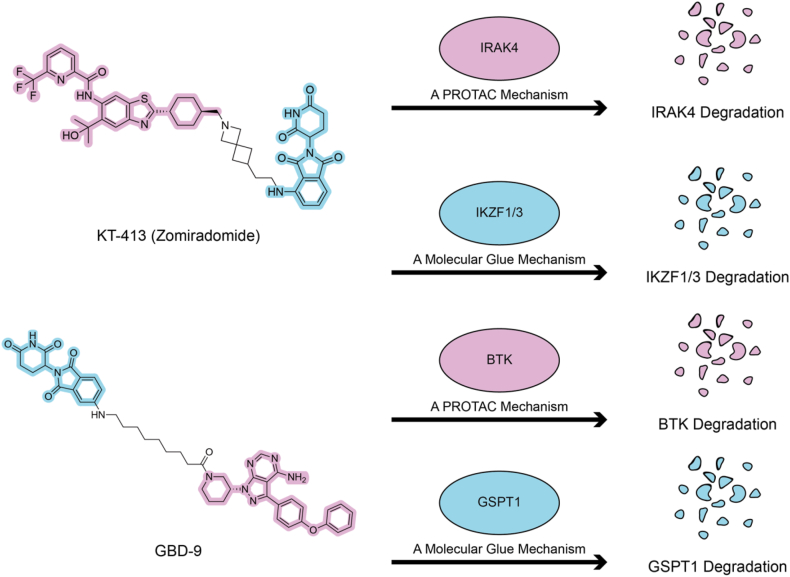
Dual-mechanism cereblon (CRBN)-based degraders that act as both PROTACs and molecular glues. KT-413 (zomiradomide) and GBD-9 are shown in the figure, each comprising a target-binding moiety (purple) linked to a CRBN-recruiting immunomodulatory imide drug (IMiD)/CC-885-derived moiety (blue). KT-413 recruits CRBN to IRAK4 to induce PROTAC-mediated IRAK4 degradation, while its IMiD core simultaneously functions as a molecular glue to degrade the CRBN neosubstrates IKZF1/3. Similarly, GBD-9 promotes PROTAC-mediated BTK degradation and molecular-glue-mediated GSPT1 degradation. Arrows denote the operative mechanism; right-hand cartoons illustrate loss of the corresponding proteins.

KT-413, developed by Kymera Therapeutics, is a clinical-stage degrader designed to treat MYD88-mutant activated B-cell-like diffuse large B-cell lymphoma (ABC-DLBCL). This molecule induces simultaneous degradation of interleukin-1 receptor-associated kinase 4 (IRAK4) and the transcription factors Ikaros (IKZF1) and Aiolos (IKZF3), which are key effectors of NF-*κ*B and type I interferon signaling, respectively. Mechanistically, KT-413 couples a high-affinity IRAK4 ligand to an IMiD-based CRBN recruiter: IRAK4 is degraded *via* a PROTAC mechanism, while IKZF1/3 are eliminated through molecular glue-induced CRBN engagement. The compound exhibits potent degradation activity in lymphoma cells (DC_50_ ≈ 4‒6 nmol/L for IRAK4; 1‒2 nmol/L for IKZF1/3) and is currently undergoing Phase I clinical evaluation^84^ (Fig. 10).

Similarly, GBD-9 is a rationally designed dual degrader that targets Bruton's tyrosine kinase (BTK) and GSPT1, which are key nodes in B-cell receptor signaling and translation termination, respectively. GBD-9 links a BTK-binding moiety to a CRBN-recruiting IMiD derivative *via* a short, optimized linker. BTK is degraded through a PROTAC mechanism, while GSPT1 is engaged and degraded *via* a molecular glue mechanism. Notably, mechanistic dissection confirmed that these degradative pathways operate independently. Specifically, blocking the BTK site with ibrutinib inhibits only BTK degradation, while CRBN inhibition abolishes both. Thus, GBD-9 achieves robust dual degradation (DC_50_ ≈ 10‒20 nmol/L) and outperforms monofunctional comparators in antiproliferative assays across multiple lymphoma models, highlighting its therapeutic potential as a prototype for multimodal degraders that simultaneously engage non-redundant oncogenic dependencies^85^ (Fig. 10).

#### 2.3.3 Dual-site/single-function CIPs

**Figure 11:**
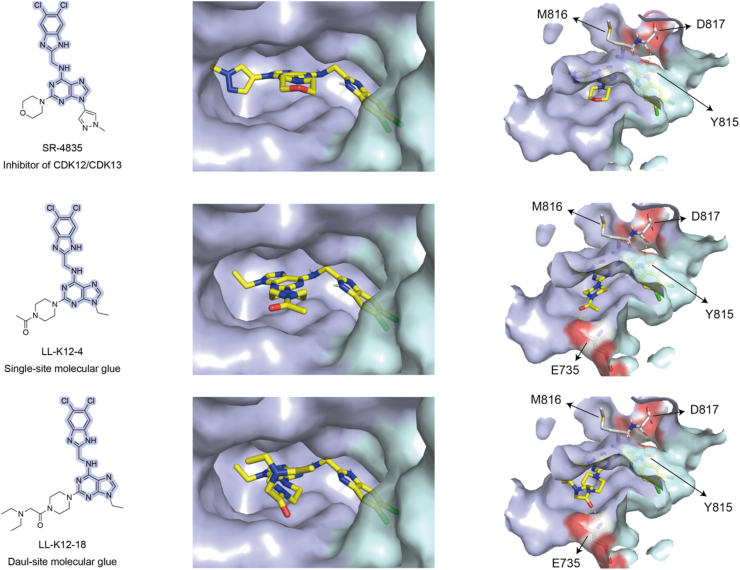
Structural snapshots of SR-4835, LL-K12-4 and LL-K12-18 reveal progressive engagement of the CDK12 ATP pocket and the DDB1 interface. Left, chemical structures highlighting the shared pyrazolo[4,3-*d*]pyrimidine core (blue). Middle, representative binding poses showing that all three analogues retain the same ATP-site anchoring mode on CDK12 (pink), while stepwise tail extension reorients the solvent-exposed substituent towards the CDK12–DDB1 contact region. Right, electrostatic surface views of DDB1 (blue) illustrating how the short acyl-piperazine tail of LL-K12-4 establishes additional contacts near the interface, whereas the di-ethyl-glycine extension in LL-K12-18 reaches an adjacent outer pocket and increases complementarity (example residues indicated), which is consistent with strengthened CDK12–DDB1 association and enhanced Cyclin K degradation potency. Representative binding poses were predicted by molecular docking using AutoDock Vina in the native ligand-binding pocket with a grid box of 15 × 15 × 15 Å. The top-ranked pose with the most negative predicted binding energy was selected for visualization. Structures were rendered in PyMOL.

Zhang et al. designed LL-K12-18, a dual-site molecular glue that enhances the CDK12-DDB1 PPI to promote the selective degradation of Cyclin K, a key regulator of DNA damage response genes^86^. Building on the structure of SR-4835 (EC_50_ ≈ 30 nmol/L in MDA-MB-231 cells)^36^^,^^72^, long-timescale molecular dynamics simulations suggested a transiently accessible pocket/groove that becomes apparent in the assembled CDK12–DDB1 context, providing an opportunity for extended substituents to engage additional interface features beyond the canonical ATP-site anchoring^86^(Fig. 11). This allosteric site is structurally regulated by the dynamic conformation of the CDK12‒DDB1 complex, and free energy calculations suggest that ligand binding at this site enhances CDK12–DDB1 interactions, thereby promoting Cyclin K degradation^86^.

A diethyl glycine moiety was introduced onto a piperazine linker to optimize molecular glue-induced PPI stabilization, enabling hydrogen bond formation with CDK12-Glu735 and DDB1-Phe949, thereby enhancing both binding affinity and degradation potency^86^. Consequently, LL-K12-18 exhibited an EC_50_ of 0.37 nmol/L, representing more than an 80-fold increase in potency compared to SR-4835^86^. Additionally, LL-K12-18 significantly enhanced the antiproliferative activity, exhibiting 88-fold and 307-fold enhancements in MDA-MB-231 and MDA-MB-468 cells, respectively^86^. Surface plasmon resonance (SPR) assays further confirmed that LL-K12-18 more effectively stabilized the CDK12–DDB1 interaction compared to single-site analogs (*e.g.*, LL-K12-4, EC_50_ = 27.42 nmol/L)^86^. Thus, LL-K12-18 effectively promotes Cyclin K degradation *via* PPI stabilization, exemplifying the potential of dual-site molecular glues in optimizing targeted protein degradation, despite its relatively weaker CDK12 kinase inhibition (IC_50_ = 283.9 nmol/L)^86^.

These case studies illustrate how binding-site engagement and functional modality can shape the activity and therapeutic potential of proximity-inducing small molecules. Compound **24** exemplifies the coupling between a single binding site and catalytic inhibition with degradation by stabilizing a productive interface, whereas YB-3-17 highlights dual-site coordination integration of target inhibition and E3 ligase recruitment to achieve a synergistic pharmacological outcome. Meanwhile, LL-K12-18 shows that selective Cyclin K degradation can be strengthened through additional interface contacts, even when kinase inhibition is relatively modest.

Notably, these strategies should be viewed as complementary design patterns that span a continuum from monovalent MGDs to more PROTAC-like hybrid architectures. However, for multivalent CIPs, which are still in early stages of development, their selectivity is often dependent on the characteristics of a specific environment and cannot be reliably predicted in advance; therefore, systematic empirical deconvolution remains crucial for determining their mechanism of action and target/off-target effects. Together, these paradigms underscore the modularity of proximity-inducing design and provide an operational framework for aligning degrader architectures with target structure, biology, and disease context (Table 2).

**Table 2:** Key multi-site and multi-function CIPs.

Strategy type	Representative compound	Binding site	Primary function
Single-site/dual-function CIPs	Compound **24**	1 site (ATP pocket)	CDK 12 inhibition & Cyclin K degradation
	(*R*)-CR8	1 site (ATP pocket of CDK 12)	CDK12 inhibition & Cyclin K degradation
Dual-site/dual-function CIPs	YB-3-17	2 sites (mTOR & CRBM-GSPT1)	mTOR inhibition & GSPT1 degradation
	KT-413	2 sites (VHL-IRAK4 & CRBN-IKZF1/3)	IRAK4 degradation & IKZF1/3 degradation
	GBD-9	2 sites (CRBN-BTK & CRBN-GSPT1)	BTK degradation & GSPT1 degradation
Dual-site/single-function CIPs	LL-K12-18	2 sites (ATP pocket & Allosteric site of CDK12)	Cyclin K degradation

## 3 Specificity, safety and design optimization

Rational design has accelerated the development of MGDs; however, translating binding into selective, safe, and durable degradation remains a significant limitation. A recurrent challenge is that small chemical changes can reshuffle ternary complex geometry and cooperativity, leading to unexpected neosubstrate profiles, off-target degradation, and safety liabilities. These issues are particularly salient for ligases with broad tissue expression (*e.g.*, Cereblon), where unintended recruitment may amplify risk. Thus, in this section, we summarized several optimization approaches, which provide a practical design logic for next-generation MGDs with improved specificity, reduced toxicity, and stronger translational robustness.

### 3.1 Canonical CRBN degron motifs and beyond

CRBN is one of the most extensively characterized E3 ubiquitin ligases in the field of MGDs^65^^,^^87^^,^^88^. This ligase possesses a well-resolved structure, pharmacological tractability, and broad neosubstrate adaptability, which make it a particularly important ligase for designing selective degraders^89^. Within the CRL4‒CRBN complex, CRBN functions as the substrate receptor, orchestrating neosubstrate ubiquitination and subsequent proteasomal degradation^90^. Clinically validated CRBN-binding MGDs, such as thalidomide, lenalidomide, and pomalidomide, demonstrate the translational success of this approach. Emerging degraders targeting previously “undruggable” proteins such as VAV1 further expand their therapeutic relevance^60^. However, fine-tuning substrate specificity without compromising degradation efficiency remains a major challenge, particularly due to the risk of off-target degradation^91^.

**Figure 12:**
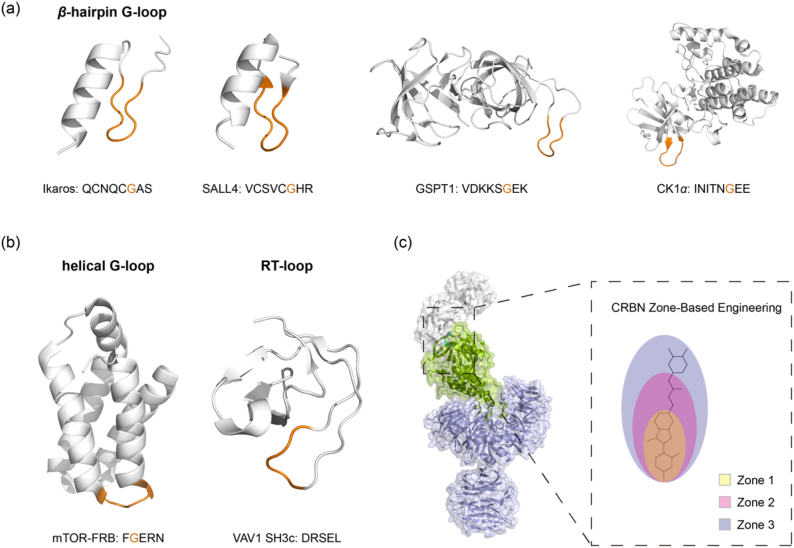
Degron motif diversity and CRBN zone-based engineering for selective molecular-glue design. (a) Canonical *β*-hairpin G-loop degrons (orange) in representative CRBN neosubstrates (IKZF1/Ikaros, SALL4, GSPT1 and CK1*α*), with the corresponding motif sequences are indicated. (b) Noncanonical CRBN-recruitment elements, including a helical G-loop-like degron within the mTOR FRB domain and an RT-loop degron within VAV1 SH3c (orange). (c) Structural overview of the CRL4-CRBN ligase (CRBN, green; DDB1-CUL4, blue) and zone-based engineering blueprint that partitions the CRBN-glue-neosubstrate interface into three optimizable regions: Zone 1 (core binding pocket, yellow) defining baseline CRBN binding, Zone 2 (proximal optimization zone, pink) tuning degron/auxiliary-contact engagement and ternary cooperativity, and Zone 3 (distal modulation zone, purple) reinforcing longer-range contacts to stabilize productive ternary complexes.

MGDs exert their effect by creating novel protein‒protein interfaces that stabilize interactions between CRBN and target proteins, collectively referred to as neosubstrates^92^. Molecular determinants of CRBN neosubstrate recognition have historically been framed around the canonical *β*-hairpin G-loop degron (Fig. 12a).

In structural terms, the canonical *β*-hairpin G-loop is typically defined as an eight-amino acid segment (G-5 to G+2) that adopts a *β*-hairpin with a tight turn and presents a key glycine (G0) within the degron core^8^. This motif interacts with CRBN through a coordinated network of hydrogen bonds and van der Waals interactions, forming a central recognition interface^17^. Proteome-wide structural surveys estimate that over 2500 human proteins possess G-loop-like motifs, highlighting the vast potential of neosubstrate space accessible to CRBN-based MGDs^65^.

A recent study by Monte Rosa Therapeutics indicates that this “G-loop rule” is more general than a strict *β*-hairpin requirement. They hypothesized that alternative folds could present an equivalent backbone geometry because CRBN engagement is largely satisfied by conserved backbone carbonyl interactions, particularly at positions G-3/G-2/G-1, that hydrogen-bond to a hotspot on CRBN (N351, H357, and W400). Consistent with this idea, relaxing the mining definition to a minimal five-residue window (G-3 to G+1), while filtering for CRBN-accessible motifs, expanded the predicted CRBN-compatible target space and uncovered a structurally differentiated “helical G-loop” class (Fig. 12b), comprising 217 matches embedded within helical segments across 184 proteins within a 1633-protein set. Representative helical G-loop candidates included the FKBP12-rapamycin binding (FRB) domain of mTOR and SRC-family kinases such as HCK, BLK, LCK, and LYN, highlighting that CRBN-compatible, G-loop-like backbone presentation can arise in both *β*-hairpin and helical contexts^25^.

Notably, the same study further showed that CRBN recruitment can extend beyond any structurally predefined G-loop-like motif *via* molecular surface mimicry. VAV1, which lacks G-loop predictions across its folded domains, was identified *via* surface-based matching to a known CRBN degron interface and engages CRBN in a compound-dependent manner through its C-terminal SH3 domain (SH3c). In this context, the apical RT-loop (residues 797‒801) constitutes a noncanonical degron element (Fig. 12b), while surrounding SH3c features, including two tryptophans (W820 and W831), provide auxiliary contacts that strengthen the productive ternary interface and help drive selectivity among VAV paralogs. RT-loop swapping and mutational analyses further establish this loop as a critical determinant of CRBN recruitment and paralog selectivity^25^.

Overall, degradation selectivity is determined by the structural and physicochemical complementarity among the MGD, CRBN, and the neosubstrate^21^. However, in practice, subtle conformational differences or insufficient ternary complex cooperativity can abrogate degradation even when binary binding is detectable^8^^,^^91^. Accordingly, modest chemical modifications to the MGD can remodel the induced interface, thereby, altering ternary complex stability and ultimately redirecting neosubstrate fate ^93^.

### 3.2 CRBN zone-based engineering

Building on the expanding repertoire of CRBN recruitment elements discussed in Section 3.1, zone-based engineering provides a structure-guided framework to modularize the CRBN-MGD-neosubstrate interface and systematically tune selectivity and degradation efficiency^65^. CRBN-based MGDs are optimized through three interaction zones to improve precision while reducing safety liabilities associated with broad ligase engagement (Fig. 12c)^65^:

Zone 1 (core binding pocket): Anchors the glue within the thalidomide-binding pocket of CRBN by engaging conserved residues (Trp380, Trp386, Trp400), thereby setting the baseline binding pose and affinity required for productive ternary complex formation.

Zone 2 (proximal optimization zone): Shapes neosubstrate selectivity by modulating the glue-exposed surface that engages degron motifs and auxiliary contacts (Section 3.1), thereby tuning ternary complex cooperativity. Substitutions at the phthalimide ring (*e.g.*, C4/C5 vectors) can shift neosubstrate profiles, as exemplified by CC-885 and ZHX-1-161.

Zone 3 (distal modulation zone): Reinforces longer-range CRBN-neosubstrate contacts beyond the immediate recognition interface to stabilize ternary complexes and promote efficient ubiquitination, as illustrated by selective GSPT1 degraders (CC-90009 and SJ6986).

Together, these zones offer a modular framework for systematically engineering CRBN-based MGDs with improved specificity, reduced off-target risk, and enhanced translational potential.

### 3.3 Multiparameter optimization

While zone-based engineering provides structural insight, several challenges regarding specificity, particularly off-target degradation, arise from multifactorial physicochemical properties not fully captured by single-parameter approaches. Multiparameter optimization (MPO), a holistic and data-driven methodology, addresses these intricacies by integrating multiple molecular descriptors.

MPO has emerged as a data-driven strategy for improving degrader specificity and safety. This method integrates multiple physicochemical and structural descriptors, and provides a holistic framework for optimizing CRBN-based degraders. A seminal study by Szewczyk et al.^94^ analyzed a library of 13,152 CRBN E3 ligase modulators (CELMoDs) and developed a tri-parametric MPO scoring system to assess and predict off-target degradation across key neosubstrates, including Aiolos, Ikaros, GSPT1, CK1*α*, and SALL4. This system prioritizes three core features: the number of aromatic carbocycles, the heteroatom counts, the fraction of *sp*^3^-hybridized carbons (F*sp*^3^).

Refinement of these parameters yields tangible safety improvements. For instance, reducing aromatic rings to fewer than two and increasing heteroatoms beyond ten significantly reduces SALL4 degradation, thereby mitigating teratogenic risk. Similarly, an F*sp*^3^ value > 0.3 correlates with reduced degradation of Ikaros and Aiolos, which may lower the likelihood of immune-related adverse effects^36^^,^^94^. Notably, this model achieved a positive predictive value (PPV) of up to 90%, demonstrating its utility in early-stage candidate triage (Table 3).

**Table 3:** Design-oriented MPO thresholds for guiding CRBN-glue neosubstrate selectivity and limiting PBMC toxicity.

Neosubstrates	SALL4	Aiolos (IKZF3)	Ikaros (IKZF1)	CK1*α*	GSPT1	PBMC (Cytotox)
Aromatic carbocycles	≥2	≥2	≥2		≥2	
Aromatic heterocycles					≤1	
Aromatic rings						≤2.8
Fraction sp^3^ C		≤0.30				
Hall-Kier alpha						≥-3.9
Heteroatoms	≤10					
Heterocycles			≤3		≤3	
Hydrogen-bond donors (HBD)		≥2	≥2			
Kappa 3 (shape)				≤4.5		
Molecular weight (MW)	≤513			≤506		≤503
Rotatable bonds				≤5.4		

Further improvements in safety can be achieved by tuning broader drug-like properties within the MPO framework. Compounds with molecular weights exceeding 506 g/mol and fewer than 5.4 rotatable bonds exhibit reduced CK1*α* degradation, likely due to steric hindrance impeding non-specific CRBN engagement^94^. Similarly, increasing the number of heterocyclic rings beyond three attenuates GSPT1-mediated cytotoxicity in peripheral blood mononuclear cells (PBMCs), further aligning MPO predictions with experimental observations^94^^,^^95^.

The conceptual foundation of MPO draws from its successful application in central nervous system (CNS) drug design^96^, where Wager et al.^97^ pioneered the consolidation of parameters like Log*P*, molecular weight, and topological polar surface area (TPSA) into unified scoring systems. In the context of MGDs, this philosophy is adapted through the integration of absorption, distribution, metabolism, excretion, and toxicity (ADMET) profiling and machine learning-driven modeling, enabling rational prioritization of degraders with favorable degradation profiles and reduced liabilities.

Thus, MPO represents a paradigm shift in molecular glue design, moving beyond intuition-driven modification toward a data-informed, multi-dimensional framework that balances efficacy, specificity, and safety. As CRBN remains a privileged E3 ligase in TPD, the continued evolution of MPO models will be critical to advancing next-generation MGDs with translational potential.

## 4 Delivery strategies for MGDs

**Figure 13:**
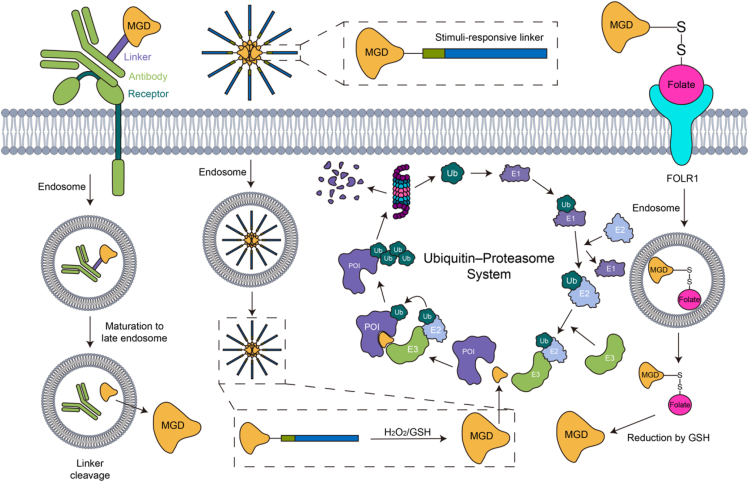
Three advanced delivery routes funnel MGDs to the ubiquitin-proteasome system. Left, antibody‒drug conjugate (ADC): an MGD (orange) is tethered to a tumor-targeting antibody (green/violet) through a cleavable linker, released after endosomal maturation, and diffuses to engage an E3 ligase. Centre, stimuli-responsive nanoparticle (NP): MGDs are embedded in a redox- or H_2_O_2_-sensitive NP; upon disassembly in the tumor endosome, payloads are freed to reach cytosolic E3 ligases. Right, folate-guided prodrug: a folate-MGD conjugate binds FOLR1, undergoes receptor-mediated endocytosis, and is reductively uncaged by intracellular GSH. All three strategies converge on the ubiquitin–proteasome cascade (center), where the liberated MGD bridges an E3 ligase (green) to the protein of interest (POI, purple), promoting polyubiquitination (Ub chains) and proteasomal degradation. Wavy black lines denote cleavable linkers; dashed insets highlight representative linker chemistries and triggers.

MGDs have emerged as a promising modality in TPD. Compared to larger heterobifunctional degraders such as PROTACs, MGDs are typically smaller and therefore often exhibit more favorable intrinsic developability. Consequently, delivery strategies for MGDs are generally not pursued as primary “PK-rescue” measures. Rather, these molecules are mainly employed to modulate biodistribution by enhancing tissue selectivity, promoting targeted accumulation, and enabling controlled local release. Notably, these features can expand the therapeutic window and reduce toxicities associated with off-tissue degradation. This section summarizes three representative approaches to enhance the clinical utility and safety profile of MGDs (Fig. 13).

### 4.1 Antibody‒drug conjugates

Antibody‒drug conjugates (ADCs) have significantly advanced targeted cancer therapies by delivering cytotoxic payloads specifically to tumor cells, thereby reducing systemic toxicity^98^, ^99^, ^100^. Despite their efficacy, traditional ADC payloads have largely been limited to agents that cause microtubule inhibition or DNA damage, which poses constraints such as limited payload diversity and emerging drug resistance^101^. However, recent innovations have expanded ADC payload options to include targeted protein degraders, notably MGDs, resulting in a novel class termed molecular glue-antibody conjugates (MACs)^102^, ^103^, ^104^, ^105^, ^106^, ^107^, ^108^, ^109^, ^110^, ^111^.

**Figure 14:**
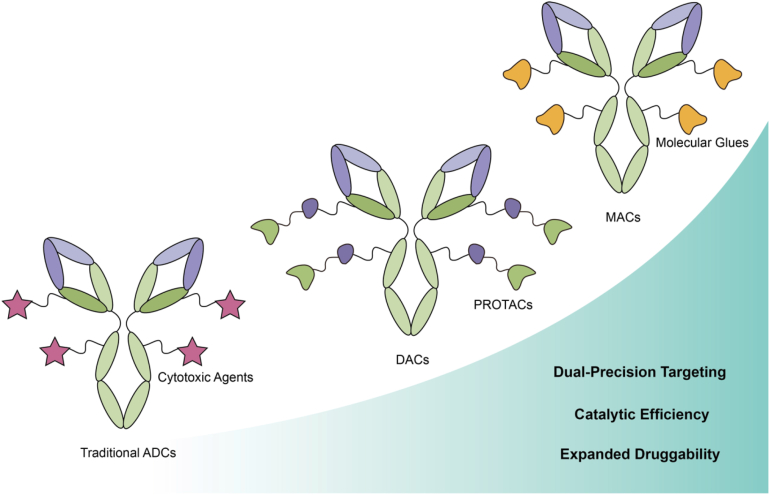
Evolution of antibody-conjugate payloads, from cytotoxic drugs to catalytic degraders. Left, a traditional antibody‒drug conjugates (ADCs) carries a cytotoxic small-molecule payload (pink star), relying on stoichiometric cell killing. Middle, a degrader‒antibody conjugate (DACs) delivers a bifunctional PROTAC (purple and green), which recruits an E3 ligase to its target protein but remains relatively bulky and less cell-permeable. Right, a molecular glue‒antibody conjugate (MACs) transports compact molecular glue degraders (yellow), enabling dual-precision targeting (antigen & molecular glue), catalytic efficiency, and expanded druggability of previously “undruggable” proteins.

MACs combine the precise targeting capability of antibodies with the catalytic, event-driven mechanism of MGDs, representing a promising evolution beyond traditional ADCs^103^. Compared with PROTAC-based degrader‒antibody conjugates (DACs), MAC payloads are typically smaller and monovalent, and often exhibit more favorable permeability and physicochemical properties, which facilitate conjugation, intracellular delivery, and targeted tissue distribution in the antibody-guided context (Fig. 14)^4^^,^^102^^,^^103^.

Several critical design considerations are essential in the development of effective MACs. First, the choice of antigen and antibody significantly impacts MAC efficacy^112^. Antigens such as HER2, CD33, and PSMA are preferred due to their consistent overexpression in cancer cells^113^, ^114^, ^115^. Notably, MAC ORM-5029, which targets HER2, has demonstrated substantial antitumor activity, even in xenograft models resistant to conventional ADC therapies, highlighting the importance of careful antigen selection^110^.

Second, linker design is paramount to MAC stability and functionality^99^. The ideal linker must securely anchor the molecular glue payload to the antibody in systemic circulation, preventing premature payload release, yet allow efficient cleavage within target cells to liberate the active degrader^99^^,^^106^^,^^116^. Commonly employed linker strategies include enzyme-sensitive cleavable linkers (*e.g.*, Cathepsin B-sensitive dipeptides like Val-Cit) or acid-labile linkers^117^^,^^118^. Innovative approaches, such as pH-responsive linkers or endosomal escape-enhancing components, can further optimize cytosolic delivery, as exemplified by Genentech's PEG-carbonate linkers^116^^,^^119^^,^^120^.

Third, payload conjugation methods critically influence MAC performance. Employing site-specific conjugation technologies to achieve a uniform drug-to-antibody ratio (DAR) significantly enhances the therapeutic index by improving potency and reducing off-target effects^121^^,^^122^. For instance, Affibody‒CR8 conjugates achieved superior specificity and safety profiles through site-specific conjugation and controlled DAR values^103^^,^^123^.

**Figure 15:**
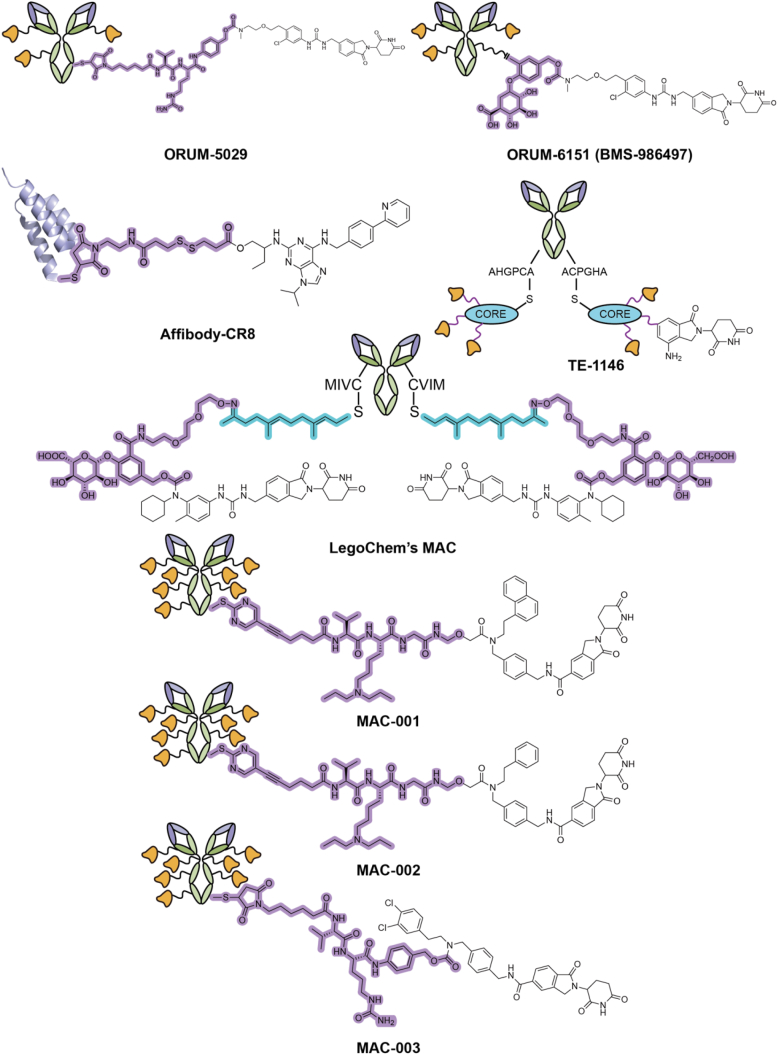
Representative molecular glue‒antibody conjugates (MACs). First-generation MACs in clinical evaluation or preclinical development. Top row: ORM-5029 (terminated; anti-HER2 × SMol006) and ORM-6151/BMS-986497 (anti-CD33 × SMol006). Middle rows: Affibody‒CR8 (anti-HER2 affibody × CR8), TE-1146 (anti-CD38 × lenalidomide), and LegoChem's site-specific EGFR-targeted MAC. Bottom rows: Medilink/Kintor's PSMA-directed c-MYC degraders MAC-001/-002/-003^103^.

Moreover, payload potency must balance catalytic efficiency with minimal off-target cytotoxicity. Molecular glue payloads generally exhibit lower inherent cytotoxicity compared to traditional ADC payloads, reducing collateral damage to healthy tissues^103^. Nevertheless, optimizing antibody internalization and intracellular trafficking, as well as payload retention within tumor cells remains essential to achieve sustained therapeutic efficacy^102^. Notably, potential resistance mechanisms such as antigen downregulation, altered proteasomal activity, or enhanced drug efflux, should also be addressed suggesting combination therapies or multi-targeted MAC strategies as potential countermeasures (Fig. 15)^102^^,^^103^.

Clinical progress has also underscored translational risks for MACs. For instance, the HER2-targeted GSPT1 programme ORM-5029 (Phase 1, NCT05511844) has been discontinued and the trial terminated following a serious adverse event (Table 4). Particularly, the sponsor cited an internal risk-benefit assessment, and public reporting has linked the decision to hepatotoxicity, including a fatal case. These observations highlight that antibody-guided delivery does not guarantee safety, because liabilities can be asset-specific and reflect antigen distribution, linker stability and release kinetics, tissue uptake of conjugates, and the intrinsic biology of the degrader payload. In contrast, the CD33-directed program ORM-6151 (BMS-986497) remains in clinical development (Table 4), suggesting that study outcomes are highly dependent on the specific antigen-payload-linker configuration rather than representing a platform-wide limitation^110^^,^^111^.

**Table 4:** Summary of the molecular glue–antibody conjugates.

MACs	Molecular glues	Antibody and linker			Clinical trail
	Target protein	Tumor-associated antigen	Linker	DAR	
ORM-5029	GSPT1	HER2	Peptide linker	4	Terminated NCT05511844
ORM-6151 (BMS-986497)	GSPT1	CD33	*β*-Glucuronic acid linker	4	Phase 1 NCT06419634
TE-1146	CK1*α* & IKZF1/3	CD38	Peptide linker	6	Preclinical
Z_HER2:342_-36_FSY_-CR8	Cyclin K	HER2	Disulfide linker	1	Preclinical
LegoChem MAC	NA	EGFR	*β*-Glucuronic acid linker	2	Preclinical
c-MYC MACs	c-MYC	PSMA	Peptide linker	8	Preclinical

Emerging MAC candidates include an EGFR-targeted MAC by LegoChem Biosciences, which exhibits substantial improvements in potency (110-fold) over unconjugated payloads and TE-1146 (CD38-Lenalidomide), thereby demonstrating robust efficacy in multiple myeloma models^124^. Additionally, innovative MAC designs targeting traditionally “undruggable” proteins such as the transcription factor, c-MYC, further expand the therapeutic scope^109^ (Fig. 15).

In conclusion, MACs represent a transformative advancement in TDP therapeutics. MACs effectively combine antibody targeting precision with catalytic molecular glue payloads, and thus have potential to overcome several limitations associated with traditional ADCs and open new therapeutic avenues in oncology. The ongoing integration of structural biology, artificial intelligence, and systematic optimization strategies is expected to accelerate the clinical translation of MACs, positioning them as cornerstone therapies for cancer treatment, despite challenges in linker optimization, payload design, and overcoming drug resistance.

### 4.2 Nanoparticle-based delivery systems

While ADCs provide a high degree of target selectivity through antibody-antigen recognition, nanoparticle-based delivery systems (NDSs) offer complementary advantages in shaping biodistribution, enhancing tumor accumulation, and enabling stimuli-responsive payload release. These systems leverage both passive targeting, *via* the enhanced permeability and retention (EPR) effect, and active targeting, through functional surface modifications, to improve intracellular delivery and minimize systemic toxicity^125^^,^^126^.

**Figure 16:**
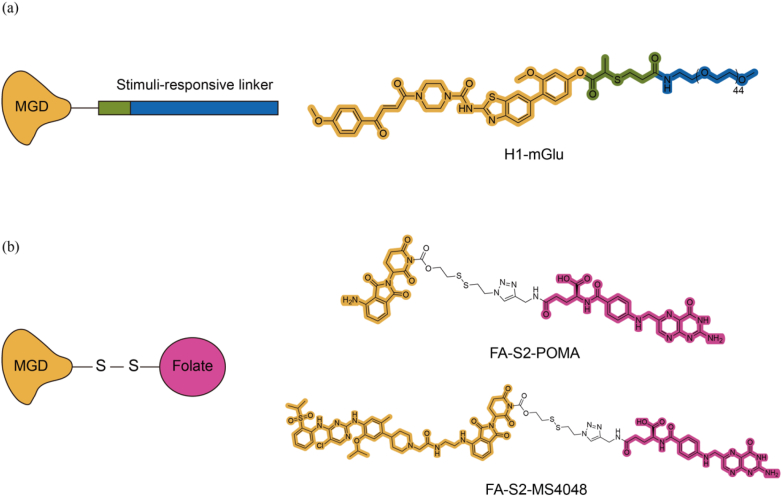
Representative chemical structures of tumor-targeted molecular glue degraders (MGDs) and their delivery conjugates. (a) Structure of H1-mGlu, a fumarate-based molecular glue conjugated to a redox-sensitive nanoparticle *via* a stimuli-responsive linker (Cle-NP platform). The linker includes a green disulfide-responsive moiety and a blue solubilizing tail to enable tumor-specific release under high glutathione (GSH) or H_2_O_2_ conditions. (b) Structures of folate-conjugated molecular glue/PROTAC prodrugs: FA-S2-POMA and FA-S2-MS4048, in which folate is attached *via* a reduction-sensitive disulfide linker to pomalidomide and MS4048, respectively. These constructs selectively activate in FOLR1-positive cancer cells to release the active CRBN-engaging payload for targeted protein degradation.

Despite significant progress in nanoparticle-enabled delivery of PROTACs, their application to MGDs has only recently begun to emerge^127^, ^128^, ^129^, ^130^, ^131^, ^132^. A seminal study by Sun et al.^53^ reported the first integration of MGDs into a self-assembled nanoparticle platform (SAN) designed for tumor-specific degradation of Bcr-Abl fusion proteins. The authors engineered a prodrug strategy wherein a newly identified fumarate-based molecular glue (H1-mGlu) was embedded into a redox-sensitive nanocarrier termed Cle-NP (Fig. 16). This system capitalized on the elevated concentrations of glutathione (GSH) and hydrogen peroxide (H_2_O_2_) in the tumor microenvironment to trigger disassembly of the nanoparticle and selective release of the active MGD at the tumor site^133^.

The *in vivo* efficacy of this delivery approach was demonstrated in K562 xenograft mouse models, where Cle-NP achieved robust degradation of endogenous Bcr-Abl/Abl proteins, leading to significant tumor regression with minimal off-target toxicity. This work represents a critical advance in the field, highlighting the feasibility and therapeutic potential of nanomaterial-enabled MGDs for cancer therapy^53^.

Beyond this pioneering example, several other nanoparticle platforms offer untapped opportunities for expanding MGD delivery, including: (1) Polymeric micelles, which offer amphiphilic environments for improved solubilization and sustained release^134^; (2) Lipid nanoparticles (LNPs), clinically validated for RNA and small molecule delivery^135^; (3) Hybrid nanocarriers, which integrate lipid-polymer systems to enhance biodistribution and payload stability^136^.

Looking forward, rationally designed multifunctional nanoparticles, such as dual-drug-loaded systems combining MGDs with immunomodulators or chemotherapeutics, may unlock synergistic antitumor responses. Likewise, tumor-penetrating peptides (TPPs) and biodegradable nanoscale frameworks may further improve tissue penetration and biosafety.

In summary, the successful implementation of Cle-NP marks a critical inflection point in the convergence of nanomedicine and targeted protein degradation. By leveraging tumor-specific triggers such as glutathione and reactive oxygen species, this platform demonstrates the feasibility of stimuli-responsive, site-selective MGD delivery *in vivo*. Moving forward, expanding MGD compatibility with diverse nanocarrier systems, such as polymeric micelles, lipid nanoparticles, and hybrid constructs, could unlock greater flexibility in tumor-selective distribution and payload formulation. Nevertheless, realizing the full translational potential of nanoparticle-enabled MGDs will require overcoming key challenges in formulation scalability, long-term stability, and regulatory pathfinding for novel degraders and nanomaterials. Addressing these barriers through integrated design frameworks will be essential for advancing MGDs toward clinical precision oncology.

### 4.3 Folate-mediated delivery system

Folate receptor alpha (FOLR1) is a glycosylphosphatidylinositol-anchored membrane protein that is overexpressed in a range of malignancies, including ovarian, breast, and lung cancers, as well as multiple myeloma, while remaining largely absent in normal tissues^137^, ^138^, ^139^. This differential expression has rendered FOLR1 a valuable molecular target for tumor-selective drug delivery^140^.

Folate (vitamin B9) is a small molecule with high affinity for FOLR1 and is internalized *via* receptor-mediated endocytosis. Compared to antibodies, folate-based targeting offers several advantages: lower immunogenicity, ease of synthesis, and improved penetration into solid tumors. These features have driven recent interest in adapting folate conjugation strategies to deliver protein degraders, such as MGDs and PROTACs, with improved tumor selectivity and reduced off-target toxicity^140^^,^^141^.

A representative example is FA-S2-POMA, a folate-caged prodrug of pomalidomide. Chen et al. designed this construct by attaching folate to the N-imide position of pomalidomide *via* a reduction-sensitive disulfide linker^142^. This design blocks CRBN binding extracellularly while allowing intracellular activation: once inside FOLR1-positive cells, elevated glutathione (GSH) levels reduce the disulfide bond, releasing active pomalidomide that engages CRBN and induces degradation of neosubstrates such as IKZF1 and IKZF3. *In vitro* studies confirmed that FA-S2-POMA selectively degraded IKZF3 in FOLR1-expressing MM.1S and SU-DHL-1 cells but showed negligible activity in FOLR1-negative THP-1 cells. The effect was abolished by free folic acid competition, CRBN knockout, or proteasome inhibition, confirming mechanism specificity^142^.

FA-S2-MS4048, a folate-conjugated PROTAC targeting ALK fusion oncoproteins was developed to demonstrate platform generalizability. The construct uses a similar disulfide linker strategy, and selectively degrades NPM-ALK and EML4-ALK in FOLR1-positive lymphoma and NSCLC cell lines (*e.g.*, SU-DHL-1, NCI-H2228, and H3122), while sparing FOLR1-negative cells. This degradation is dependent on CRBN, FOLR1, and intracellular redox activity, affirming the modularity of this delivery approach^142^.

Together, these findings highlight folate-guided degradation as a promising strategy for tissue-selective delivery of both MGDs and PROTACs. Folate conjugation couples small-molecule degraders to a clinically validated targeting ligand and exploits tumor-specific redox environments for controlled release, thereby enhancing therapeutic precision and minimizing systemic exposure. Future innovations in linker chemistry, payload design, and formulation may further expand the translational potential of folate-mediated degrader delivery in precision oncology.

### 4.4 Summary and perspectives on delivery strategies

Effective delivery strategies have emerged as critical determinants in MGDs from promising laboratory innovations to viable clinical therapies. Although rational compound design and neosubstrate specificity remain foundational, optimal clinical outcomes often depend on achieving targeted tissue distribution and controlled intracellular release of active MGDs, thereby improving the therapeutic index and reducing off-tissue degradation. The primary modalities discussed herein, antibody‒drug conjugates, nanoparticle-based platforms, and folate-mediated systems, collectively aim to engineer biodistribution, limit systemic off-tissue exposure, and improve on-target therapeutic efficacy.

Each delivery modality presents unique strengths and specific challenges. ADCs leverage highly selective antibody–antigen interactions, backed by considerable clinical experience; however, they must address issues of linker stability, drug-to-antibody ratio (DAR) consistency, and efficient intracellular payload release^102^^,^^103^. Nanoparticle systems offer versatile solutions, allowing for precise spatiotemporal drug release through stimuli-responsive materials and enabling multifunctional payload integration^53^. Nevertheless, their clinical translation demands overcoming batch-to-batch variability and optimizing endosomal escape mechanisms. Folate-mediated approaches exploit receptor-mediated endocytosis and possess advantages such as minimal immunogenicity and small molecular size, making them particularly suited for solid tumors, although competition with physiological folate and receptor heterogeneity may pose practical limitations^142^.

Future progress will likely emerge from integrating these approaches into hybrid delivery systems that capitalize on their complementary strengths. For example, folate-decorated, stimuli-responsive nanoparticles or antibody-guided nanocarriers could provide synergistic benefits, including enhanced tumor selectivity, controlled payload release, and improved pharmacodynamics tailored specifically to MGDs. Advances in linker technologies, E3 ligase selectivity, and dual-payload constructs will further optimize these hybrid systems, balancing payload stability with precise intracellular release kinetics.

Critical hurdles remain, including scalable manufacturing processes to ensure product uniformity, quantitative benchmarks for evaluating endosomal escape efficiency, and comprehensive immunogenicity assessments to mitigate potential adverse reactions upon repeated administration. Additionally, aligning novel MGD delivery platforms with current regulatory frameworks will be essential for their successful clinical translation.

Ultimately, refining delivery technologies will transform current limitations into strategic opportunities, accelerating the clinical realization of MGDs as powerful, targeted cancer therapeutics.

## 5 Conclusions

MGDs have emerged as transformative tools in TPD, offering an innovative means of selectively eliminating disease-associated proteins, particularly those traditionally deemed undruggable. This review has highlighted three core dimensions critical to advancing MGDs: rational design strategies, specificity optimization, and advanced delivery platforms. Together, these pillars underscore the considerable promise of MGDs as a next-generation therapeutic class.

First, rational design has fundamentally shifted MGD development from serendipitous discoveries towards precise, chemically sophisticated platforms. Strategies including covalent handle-based modifications, PPI-driven approaches, and multi-functional constructs provide enhanced control over degradation efficiency, target specificity, and substrate breadth. Additionally, integrating structure-guided design with chemoproteomics continues to expand the repertoire of recruitable E3 ligases and the range of neo-substrates, thus broadening the therapeutic potential of MGDs across diverse disease indications.

Second, despite these advancements, achieving selective and safe degradation remains a central challenge in translating MGDs into clinical applications. In this regard, emerging methods such as CRBN degron motifs, zone-based engineering and MPO have markedly advanced our ability to fine-tune MGD specificity while minimizing off-target liabilities. These approaches promise degraders with more predictable pharmacological profiles, potentially enhancing their therapeutic indices and clinical utility.

Third, the clinical feasibility of MGDs increasingly depends on delivery systems that can shape biodistribution and enable targeted distribution and controlled intracellular release, thereby improving the therapeutic index and reducing off-tissue degradation. ADCs, NP-based carriers, and folate-mediated delivery methods exemplify how targeting-enabled platforms can concentrate active MGDs within diseased tissues, facilitate tissue-specific degradation, and mitigate systemic off-tissue exposure, thereby accelerating the development of MGDs for precision oncology.

In summary, the synergy between rational design, enhanced selectivity, and innovative delivery solutions is propelling MGDs toward clinical translation. However, future advancements in this field will increasingly rely on the integration of computational and data-driven methodologies. Artificial intelligence and machine learning have the potential to profoundly influence all phases of MGD development, from computational candidate generation and ternary complex modeling to multiparametric optimization and formulation design. These technologies offer powerful tools to refine degradation efficiency, reduce off-target effects, and enable personalized therapeutic strategies through predictive modeling and exposure-biodistribution simulations. The field stands poised to deliver next-generation MGDs that are safe, selective, and tailored for precision medicine, by embracing such innovations, along with ongoing progress in structural biology and systems pharmacology.

## Author contributions

Lieen Ma drafted the initial version of the manuscript and prepared all figures and tables. Ning Wang, Jingjing Zhu, Shan He and Bin Zhang provided guidance on the conceptualization and overall structure of the manuscript, and critically reviewed and revised the text. Lingjie Wu carefully checked the language, formatting and other textual details. All authors read and approved the final manuscript.

## Conflicts of interest

The authors declare no conflict of interests.
